# A Novel Non-Rodent Animal Model of Hydrochloric Acid-Induced acute and chronic lung injury.

**DOI:** 10.21203/rs.3.rs-4758497/v1

**Published:** 2024-08-13

**Authors:** Pavel A. Solopov, Ruben Manuel Luciano Colunga Biancatelli, Tierney Day, Christiana Dimitropoulou, John D Catravas

## Abstract

Hydrochloric acid is one of the most prevalent and dangerous chemicals. Accidental spills occur in industrial plants or during transportation. Exposure to HCl can induce severe health impairment, including acute and chronic pulmonary diseases. We have previously described the molecular, structural, and functional aspects of the development of chronic lung injury and pulmonary fibrosis caused by intratracheal instillation of HCl in mice. Although mouse models of human disease have many advantages, rodents are evolutionary far from human and exhibit significant anatomical and physiological differences. Genetic and anatomic similarities between rabbits and humans are significantly higher. Rabbit models of HCl-induced lung injury have been used sparsely to evaluate acute lung injury. In this study, for the first time, we utilized rabbits as a model of HCl-induced pulmonary fibrosis and chronic lung injury. We present molecular, histological, and functional evidence that demonstrate the utility of using this model for studying new pharmaceutics against pulmonary fibrosis.

### Introduction

Acute inhalation of HCl vapors can lead to chemical pneumonitis and acute respiratory distress syndrome (ARDS), conditions characterized by immediate lung inflammation and difficulty breathing, as observed in numerous case reports [1–4]. Long-term inhalation is of particularly concern, with studies indicating an increased risk of chronic obstructive pulmonary disease (COPD) among those exposed to acid mists [5]. Chronic exposure, even to low levels of HCl, can induce progressive respiratory conditions like bronchitis and pulmonary fibrosis, suggesting a dose-response relationship between HCl concentration and respiratory morbidity [6]. The pathophysiology of HCl-induced lung injury involves direct damage to alveolar epithelium and capillary endothelium, leading to increased vascular permeability, edema, and inflammation. Chronic lung injury often results in progressive pulmonary fibrosis, characterized by the accumulation of fibroblasts and myofibroblasts, leading to excessive deposition of extracellular matrix proteins and stiffening of lung tissue [7]. These mechanisms were elucidated in rodent models utilizing mice, rats, and guinea pigs [8–12]. Despite the advantages of these models, the similarities in airway structure and inflammatory response between rabbits and humans are stronger and emphasize the utility of rabbits in studying the underlying mechanisms of lung diseases and in formulating therapeutic interventions [13][14]. The inflammatory responses exhibited by the rabbit models, particularly in cases of asthma, closely resemble those observed in humans [15]. Over the past 30 years, a number of studies have examined the early effects of hydrochloric acid exposure on the lungs of rabbits, focusing on acute lung injury [16–18].

In this study, we developed a new rabbit model for chronic lung injury and pulmonary fibrosis that includes a relatively long observation period and high survival rates. This model could be of benefit to those interested in chemical-induced lung injury and drug discovery for pulmonary fibrosis.

### Materials and Methods

## Materials

Hydrochloric acid (37%), ACS grade, methacholine chloride USP grade, radioimmunoprecipitation assay (RIPA) buffer, and protease inhibitor cocktail were obtained from Sigma-Aldrich Corporation (St. Louis, MO, United States). AnaSed (xylazine) USP grade, Ketaset (ketamine) USP grade and Buprenorphine were obtained from Covetrus (Portland, ME, United States). Formaldehyde ACS reagent, 37%, was purchased from ThermoFisher Scientific (Waltham, MA, United States), the BCA Protein assay kit from Pierce Co. (Rockford, IL, United States), and EDTA from GE Healthcare (Chicago, IL, United States). CD163 and HSP90 antibodies were obtained from ThermoFisher Scientific (Waltham, MA, United States).

## Animals and treatment groups

New Zealand White rabbits, both male and female, weighing 1.5–2.5 kg, were anesthetized intramuscularly with ketamine (20–50 mg/kg) and xylazine (3–5 mg/kg). After confirming adequate anesthesia through the absence of pain reflexes, the animal was placed in dorsal recumbency. The neck was shaved and washed twice with alcohol. A single subcutaneous (sc) injection of buprenorphine (0.12 mg/kg) for analgesia was administered. This was followed by a 10–12 ml/kg sterile saline bolus administered subcutaneously to hydrate the animal, and exposure to 1.5L/min of 100% oxygen for 10 minutes. A 25G needle was then inserted between consecutive tracheal rings to thread a 2-inch-long plastic catheter through it; after which, the needle was retracted. Saline (1.5 ml/kg) or 0.1N HCl (1, 1.5, or 2 ml/kg) was rapidly injected through the catheter and flushed with 4 ml/kg of air. The catheter and needle were then removed, and the rabbit was placed in sternal recumbency and administered 100% oxygen via a face mask. The oxygen flow was reduced by 0.5L/min approximately every 10–15 minutes. Once on room air and maintaining an $SPO_2$ saturation above 90% for approximately 10 minutes, the animal was moved from the procedure table to the recovery cage.

Rabbits were randomly divided into six treatment groups: 1) male rabbits exposed to normal saline (VEH); 2) male rabbits exposed to 0.1N HCl at a dose of 1 ml/kg; 3) male rabbits exposed to 0.1N HCl at a dose of 1.5 ml/kg; 4) male rabbits exposed to 0.1N HCl at a dose of 2 ml/kg. The animals underwent bronchoalveolar lavage fluid (BALF) collection, lung tissue analysis (Western blotting, real-time qPCR), lung function measurements (FlexiVent), and histological analysis at 4-, 10-, and 60-days post-exposure. Due to high mortality rates, histological studies, BALF, and lung tissue analyses for group 4 were conducted only at 60 days post-exposure for ethical reasons. Female rabbits were included in the study after optimizing the HCl doses; they were exposed to VEH (group 5) or 1.5 ml/kg of 0.1N HCl (group 6). The experimental design is illustrated in Fig. 1.

## HCl dose calculation and translation to human exposure

The doses 1, 1.5 and 2 ml/Kg of 0.1N HCl correspond to 3.58, 5.38, and 7.17 mg/Kg respectively, in rabbits and to 1.19, 1.79 and 2.39 mg/Kg in humans. The human deposited dose was calculated using Eq. (1) as previously described [19]:

$$X_h = X_a \left(\frac{M_a}{M_h}\right)^{(1-b)}$$

1

Where, $X_h$ = human drug dose normalized to body mass (μg/Kg); $X_a$ = animal drug dose per unit body mass (3.58, 5.38 or 7.17 mg/Kg); $M_a$ = animal body mass (2.5 Kg); $M_h$ = human body mass (70 Kg); $b$ = 0.67 for inhaled translation model.

There is no accurate analysis of dose-responses to HCl inhalation in humans. Henderson and Haggard [20], however, reviewed and classified HCl gas levels based on concentration, exposure time, and their related symptoms in humans, defining as "dangerous and life-threatening" a brief exposure at 2000 ppm. Considering 10 to 25 minutes as the essential time in the "real world" for the personnel to clear the area after a spill of HCl gas, using Eq. (2), as proposed in [21], the corresponding deposited doses are:

$$Deposited_{Dose} = (C \times RMV \times D \times IF \times DF) \div BW$$

2

Where, C: concentration of substance in the air (2000 ppm – 2.980 mg $L^{-1}$); RMV: respiratory minute volume (6 L $min^{-1}$); D: duration of exposure (10, 20 and 25 min); BW: body weight (70 Kg); IF: inhalable fraction (is assumed to be 100% for Mass Median Aerodynamic Diameter (MMAD) less than 3–4 μm); DF: deposited fraction. Deposited fraction was assumed to be 40% in humans [22]. Thus, for 10, 20 and 25 min exposure of humans to 2000ppm HCl, the deposited amounts are:

$Deposited_{Dose}$ (10 min) = 1.02 mg/Kg

$Deposited_{Dose}$ (20 min) = 2.04 mg/Kg

$Deposited_{Dose}$ (25 min) = 2.55 mg/Kg

These values are remarkably close to the deposited doses calculated in our rabbit model of HCl exposure.

## Bronchoalveolar Lavage Fluid (BALF) White Blood Cell Number and Total Protein Concentration

BALF collection involved the instillation and subsequent retrieval of sterile 1x PBS (10 mL) through a syringe cannula connected with 1000 μL pipette tip, which correspond to the diameter of the rabbit's trachea. The number of white blood cells (WBC) in the fluid was measured using a hemocytometer. Following centrifugation of the fluid at 2500× g for 10 minutes, the supernatant was harvested to measure protein levels. The concentration of total protein was assessed using a micro bicinchoninic acid (BCA) Protein Assay Kit, adhering to the guidelines provided by the supplier.

## Histopathology, Immunohistochemistry and Lung Fibrosis Scoring

The animals were euthanized with 120 mg/kg pentobarbital i.v., their chests were opened, and the lungs were fixed in 10% formaldehyde. Mid-transverse sections were cut from the formalin-fixed lung tissues and embedded in paraffin. To identify the lung lobes, most involved in the pathologic process, every lobe was analyzed. 5 μm thick sections were obtained from these blocks, and stained with Masson's trichrome stain, anti-HSP90β, and anti-CD163 monoclonal antibodies. Twenty fields on each slide were randomly selected and examined under 20× and 40× magnifications. An investigator, unaware of the group identities, used the Lung Injury and Ashcroft scoring methods to evaluate all trichrome-stained slides for the degree of lung injury and pulmonary fibrosis [23, 24].

## Lung Tissue Collection

Immediately after euthanasia, the lungs were flushed with saline with EDTA through the pulmonary circulation, dissected from the thorax, and the left caudal lobes were snap-frozen, and prepared for subsequent analysis.

## RNA isolation and Quantitative Real-Time PCR (qPCR)

Lung tissue, stored in an RNAlater solution, was dried and homogenized in TRIzol®, followed by a cleaning-up step using the RNeasy Mini Kit. The purified RNA was transcribed into cDNA using the SuperScriptTM IV VILO Reverse transcriptase Kit and was analyzed by real-time qPCR with SYBR Green Master Mix on a StepOne Plus Real-Time PCR System (Applied Biosystems v.2.3). The results were evaluated using the standard curve method and were expressed as the fold of the control values, normalized to β-actin. Specifically designed primer pairs and qPCR conditions were applied to selectively determine the expression of rabbit β-actin, collagen 1α2.

## Lung Mechanics Measurements

The rabbits were anesthetized with ketamine 50mg/kg i.m., tracheostomized with a plastic 1000 μL micropipette tip, and connected to a FlexiVent small animal ventilator equipped with FX6 module for rabbits (SCIREQ Inc., Montreal, QC, Canada). Ventilation was performed at a tidal volume of 10 mL/kg and a respiratory rate of 150/min. A 15-minute stabilization period was allowed before measurements began. Initially, resting static compliance (Cst, mean of three values) and pressure-volume (PV) loops were assessed by incrementally increasing airway pressure to 30 cm $H_2O$ and then decreasing it, to assess the lungs' intrinsic elasticity. In fibrosis, Cst decreases, and PV curves shift rightwards. Subsequently, Snapshot-150 and Quick Prime-3 maneuvers were executed. Respiratory system resistance (Rrs), elastance (Ers), Newtonian resistance (Rn), tissue damping (G), inspiratory capacity (A), and PV loop curvature (K) were calculated and averaged over 12 recordings. After the study, the rabbits were euthanized with 120 mg/kg pentobarbital i.v.

## Statistical Analysis

The data are shown as means ± standard error of the mean. The statistical significance of differences among groups was assessed using one- or two-way analysis of variance (ANOVA), accompanied by Tukey's or Bonferroni's post-hoc tests. Statistical evaluations were conducted using GraphPad Prism v. 9.0 (GraphPad Software, San Diego, CA, USA). A p-value of less than 0.05 indicated significant differences between groups.

### Results

## HCl dose response.

Rabbits instilled with 2 ml/kg of HCl exhibited 60% survival, whereas 100% of those exposed to either 1.5 or 1 ml/kg survived during the 60 days of post-exposure observation (Fig. 2A). Lung sections taken 10 days post-exposure and stained with Masson's trichrome (Fig. 2B), along with BALF cellularity assessments (Fig. 2C), indicated that the lower dose (1 ml/kg) was not effective enough to elicit a strong inflammatory response leading to chronic lung injury. Based on this data and adhering to ethical principles, a dose of 1.5 ml/kg of 0.1 N HCl was chosen for subsequent studies.

## Evidence of HCl-induced alveolar inflammation and endothelial/epithelial dysfunction.

Alveolar inflammation and endothelial/epithelial barrier dysfunction occur on the fourth day post 1.5 ml/kg (0.1N) HCl exposure and remain elevated until day 60 compared to controls (Fig. 3). The content of alveolar immune cells and total protein in BALF is 25- and 3-fold higher, respectively, 4 days post-HCl intratracheal instillation compared to rabbits administered saline. At day 10, both parameters slightly decrease. The elevated levels of total protein at day 60 in the BALF indicate persistent endothelial/epithelial permeability in the chronic phase of lung injury.

## Histological evidence of HCl-induced lung injury and pulmonary fibrosis.

Histological studies were conducted at 4, 10, and 60 days after intratracheal injection of 0.1N HCl, as well as at 60 days following injection of sterile saline as a control. Signs of severe alveolar inflammation were confirmed by the substantial presence of alveolar inflammatory cells, predominantly neutrophils and monocytes, observed 4 days post HCl injection, compared to control rabbits. Collagen fibers had already formed and lined the surfaces of the alveoli (Fig. 4A). By day 10, significant migration of immune cells to the interstitial areas was observed, impacting the architecture of lung parenchyma. Rabbits that received HCl intratracheally and were euthanized 60 days post-exposure showed a reduction in alveolar spaces, complete fibrous obliteration in the caudal lobes, and areas of granuloma formation. Since the cranial lung lobes were more damaged by HCl exposure than the caudal ones due to their anatomical position, all subsequent molecular analyses were performed using cranial lobes. All pathohistological changes were scored by a pathologist (Fig. 4C, D).

## HCl-induced activation of pro-fibrotic signaling pathways.

We examined the hemoglobin scavenger receptor CD163, a marker of macrophage type 2 (M2) activation. Lung sections were immunostained for CD163 at 10- and 60-days post-instillation. The expression of CD163 was significantly higher in rabbits treated with HCl at day 10 compared to control animals, as well as to samples taken 60 days after HCl instillation. Fibroblast activation occurred in areas of the lung with pronounced edema (Fig. 5A, B). Immunohistochemical detection of HSP90 in lungs displayed differences between saline- and HCl-instilled animals. Within the control group, HSP90 stained only few cells (1–3 cell per 20 X field) while interalveolar, interstitial, and perivascular spaces were free from the protein stain. Conversely, rabbits instilled with HCl showed a dramatic increase in the immunostaining of HSP90 (Fig. 5C, D). High levels of HSP90 were found at both 10 and 60 days and high concentrations were detected near macrophages. HSP90 at 10 days was mainly concentrated in the perialveolar regions, in areas with large assembly of macrophages but did not involve spaces invaded by neutrophils. At 60 days, HSP90 stained completely the fibrotic lung and displayed the strongest concentration in the peribronchial and perivascular areas. Collagen 1α2 and Fibronectin, well-known extracellular matrix protein, whose expression increases in Pulmonary Fibrosis also increased at 60 days after HCl exposure, compared to Saline-instilled controls (Fig. 5E, F).

## Evidence of HCl-induced respiratory dysfunction.

Rabbits exposed to HCl exhibited severe pulmonary dysfunction at days 4 and 10 compared to saline-treated rabbits. The pressure-volume (PV) loops and static compliance (Cst) showed a significant downward shift compared to control rabbits, while respiratory resistance (Rrs) and elastance were markedly increased in the acute phase of inflammation (Fig. 6). Although most parameters returned to their physiological norms by day 60, PV loop and Rrs remained elevated, indicating chronic lung injury.

## Sex differences in the rabbit model of HCl-induced chronic lung injury and pulmonary fibrosis.

Female rabbits were administered the same dose of HCl as male rabbits. Similarly to our previous studies that showed sex differences in chemically induced lung injury in mouse models, female rabbits given HCl intratracheally exhibited less alveolar inflammation, edema, and no fibrous obliteration areas compared to their male counterparts (Fig. 7). However, there was still significant elevation of immune cells and collagen deposition in lungs compare to control female rabbits. The lung function studies also confirmed that following HCl exposure, male subjects develop more severe respiratory disfunction compared to females, as reflected in PV-loops and Rrs values.

### Discussion

Pulmonary fibrosis is characterized by the progressive scarring of lung tissue that ultimately compromises pulmonary function [25]. Despite extensive studies, the pathophysiological mechanisms underlying this condition remain incompletely understood, hindering the development of effective treatments [26]. Animal models have been instrumental in elucidating these mechanisms, offering valuable insights into the disease's progression and potential therapeutic interventions [27]. Among these models, the new rabbit model of hydrochloric acid-induced pulmonary fibrosis could be a pivotal tool for studying the disease's etiology and testing novel treatments. The rabbit, being phylogenetically closer to humans than rodents and sharing anatomical, physiological, genetic, and biochemical similarities, is preferentially used in studies of pulmonary, cardiovascular, and metabolic conditions, including airway obstructive disease, embolic stroke, arteriosclerosis, and cystic fibrosis, as well as for drug screening, antibody production, and therapeutic protein production [15]. Using rabbit models to study acute and chronic lung injuries offers several advantages compared to rodent (such as mice or rats) models, primarily due to differences in size, physiology, and immune responses. Rabbits are larger than rodents, which makes surgical interventions, intratracheal administrations, and detailed physiological monitoring more feasible [13]. Their bronchial branching patterns and the distribution of airway receptors, is more similar to humans, making them a more relevant model for studying airway diseases and evaluating the efficacy of inhalation therapies [28]. Rabbits exhibit immune responses that are often more comparable to those of humans, particularly in terms of antibody production, immune cell profiles, and the response to infectious agents and inflammatory mediators [29]. Similarity, this can provide more accurate insights into the pathogenesis and progression of lung diseases, as well as the potential efficacy and safety of therapeutic agents. The mechanisms of disease progression and tissue repair in rabbits closely mirror those in humans, especially for fibrotic lung diseases [15]. In agreement, our pathohistological data demonstrate that rabbits naturally develop more pronounced and clinically relevant fibrotic responses after injury, which can be crucial for studying the efficacy of antifibrotic therapies. The pharmacokinetic and pharmacodynamic profiles of drugs in rabbits more closely resemble those in humans compared to smaller rodents [30]. This similarity can lead to more accurate predictions of drug dosages, efficacy, and side-effect profiles in humans. Despite these advantages, there is still several limitations. Unlike rabbits, humans have respiratory bronchioles, which evidently contribute to the development of emphysema and fibrosis [31]. Rabbit mono- and polyclonal antibodies are the most popular and have been widely utilized as analytical tools in biomedical research, particularly for immunological studies [32]; this makes in-vitro and in-vivo studies in rabbits more complicated due to the lack of suitable commercial antibodies from other species.

Previous studies in rabbits employed higher doses and concentrations of HCl and focused on the acute outcomes of HCl exposure. An earlier study (1976) used HCl 1.5 pH at 2 ml/kg that produced 100% mortality 4 h post injection [33]. Several others used 3-5ml/kg doses with similarly high mortality rates by 4-14hrs [18][16][17]. In our previous study of HCl-induced chronic lung injury in mice, we identified 0.1N HCl as most effective to induce pulmonary fibrosis [10]. In this study we tested 3 different doses of 0.1N HCl (1, 1,5 and 2 ml/kg) to identify the dose sufficient to cause chronic lung injury in rabbits with minimal mortality. Similarly to previous studies studies, the higher dose of HCl (2ml/kg) provoked high mortality, while the lower dose did not cause significant alveolar damage. The chosen optimal dose to study HCl-induced pulmonary fibrosis in rabbits was 1.5 ml/kg of 0.1N HCl. Our calculations, largely discussed in the methods section, confirm the clinical translatability of our model to humans.

The effects of HCl on BALF composition has been reported previously in rabbits. Intratracheal administration of HCl to rabbits resulted in a significant increase in the number of neutrophils and a decrease in alveolar macrophage activity in BALF [18]. In another study, rabbits instilled intratracheally with HCl demonstrated an increase in extravascular plasma 6–24 h post exposure [34]. In our previously published HCl-induced mouse lung injury model, we observed that the immune cell content and total protein concentration in BALF peak at day 4 post-instillation, decrease sharply by day 10, and stabilize at that level until day 30, when the injury transitions to a chronic form [10]. Based on our data and symptoms from medical case reports [2], we can infer that the most intense interval of acute lung injury occurs at 4 days after exposure. At the same time, both the cellularity and increased protein remain elevated at 60 days, which could reflect chronic lung injury and developing pulmonary fibrosis.

Pulmonary fibrosis in humans is characterized by the overexpression of ECM and the formation of scar tissue. X-ray images of IPF patients typically show net-like curvilinear opacities in a bilateral, asymmetric pattern, primarily in the caudal lobes [35]. In our previous studies of chemically induced lung injury in mice, we observed that collagen deposition and fibrous obliteration of lung parenchyma were equally spread across all lobes with damaged areas in the center of the lobes and slightly involved stromal areas [10, 36, 37]. Studies utilizing the bleomycin induced PF mouse model also demonstrated the spread of damaged areas throughout the lungs. Here we see that injury is not uniform and that the lobes most involved into the fibrogenesis are cranial (Fig. 4B). We speculate that the anatomical position of lung lobes in human and other animal species plays an important role in the pathohistological picture of fibrotic lung. Due to gravity, most of the exudated lung fluid tends to penetrate to the lower lung tissues relative to physiological postures. In our mouse model of HCl-induced pulmonary fibrosis, we observed moderate alveolar thickening with some areas with altered parenchymal architecture by day 10 and fibrosis within 30 days [10]. Since pulmonary fibrosis is usually diagnosed 1-2.7 years after the initial respiratory diagnosis, it is difficult to determine the exact time it takes for the first lung tissue scar to develop, but the condition generally becomes more severe over time in most patients [38, 39]. In this rabbit model, we observe change of parenchymal architecture at day 10 and fibrotic outcomes within 60 days. Rodents have faster metabolism, while rabbit’s metabolic pattern is almost similar to the human [40].

Fibroblast and macrophage activation play important roles in the pathogenesis of fibrosis [41]. Accumulation of CD163 + and CD204 + macrophages in lungs is associated with worse clinical outcomes in IPF patients [42]. The receptor CD163 is a macrophage activation marker, with serum levels rising in several acute inflammatory states and some fibrosing diseases and monocyte-derived macrophages differentiated by macrophage colony-stimulating factors contributing to the pathophysiology of idiopathic pulmonary fibrosis [43]. CD163 expression in BALF alveolar macrophages is higher in the IPF patients compared to both healthy controls and lung cancer patients [44]. In a study employing CD204 knockout mice, Beamer and Holear suggested that the functions of CD204 are crucial for the development of fibrosis and the resolution of inflammation [45]. Nouno et al. reported that the levels of CD163 in lung biopsy samples from IPF patients were significantly elevated compared to control patients, whereas CD204 + cell counts did not differ [42]. Here, in rabbits, we see the dramatic expression of CD163 in alveolar spaces occupied by macrophages, reflecting a closer similarity of humans to the rabbit model compared to the mice model.

Sontake and colleagues identified the role of heat shock protein 90 (HSP90) in the regulation of IPF-related fibroblast activation [46]. We previously reported the activation of HSP90 in mouse lungs, and in human lung microvascular endothelial cells exposed to HCl [10, 47, 48]. In agreement with these investigations, in this study we observed overexpression of HSP90 in HCl-exposed lung tissue.

Respiratory disfunction is the major clinical outcome of both acute lung injury and pulmonary fibrosis. IPF patients exhibit severe changes in lung dynamics [49]. We previously reported that HCl-exposed mice demonstrate rightward shift of PV-loops, increased respiratory resistance and elastance and airway hyperreactivity in response to methacholine at day 30 post exposure [10, 36, 47, 50]. Here, for the first time, we employed FlexiVent Model 6 to test the lung mechanics dysfunction in rabbits and observed similar pathologies in rabbits injected intratracheally with HCl at 4, 10 and 60 days post exposure.

It is well-known that interstitial lung diseases, including pulmonary fibrosis, exhibit sexual dimorphism [51]. Clinical data indicate that IPF predominantly affects males, which may be attributed to differences in exposure history, the expression of X-chromosome-related inflammatory genes, and the influence of sex hormones [52, 53]. We previously showed histological, molecular, and functional difference between male and female mice, exposed to HCl [54]. We also demonstrated that those differences are related to the protection effects of estrogens [36]. Our current study also showed persistent histological, molecular, and functional differences between male and female rabbits. As we expected, female rabbits did not develop as severe fibrosis as males. The mechanisms underlying the sexual dimorphism in HCl-induced pulmonary fibrosis in rabbits require further investigation.

In conclusion, this model, which induces fibrosis through direct HCl injury, replicates several key aspects of human pulmonary fibrosis, including the inflammatory response, fibroblast proliferation, and extracellular matrix deposition. Despite the problem of limited availability of commercial antibodies for rabbits, this model is potentially more relevant to humans and can be used to test new antifibrotic pharmaceuticals.

### Declarations

## Ethical Approval

Animal studies were approved by the Institutional Animal Care and Use Committee (IACUC) of Old Dominion University (Protocol #23 – 011). They abide by the principles of animal experimentation as published by the American Physiological Society, and were carried out in Animal Biosafety Level 2 (ABSL-2) facility at the Frank Reidy Research Center for Bioelectrics, ODU, Norfolk, VA. All pain-related procedures (anesthesia, HCl injection, lung mechanics tests and euthanasia) were carried out in the presence of a licensed veterinarian and a member of IACUC.

## Consent for Publication

Not applicable

## Funding

Supported by the CounterACT Program, National Institute of Health Office of the Director (NIH OD), and the National Institute of Environmental Sciences (NIEHS), grant number U01ES030674.

## Author Contribution

Conceptualization, J.D.C.; methodology, P.S., R.M.L.C.B., C.D., and J.D.C.; validation, P.S., R.M.L.C.B., C.D., and J.D.C.; formal analysis, P.S., R.M.L.C.B., T.D., and C.D.; investigation, P.S., R.M.L.C.B., T.D., and C.D.; resources, J.D.C.; data curation, P.S., R.M.L.C.B. writing—original draft preparation, P.S. and R.M.L.C.B.; writing—review and editing, P.S., R.M.L.C.B., and J.D.C.; supervision, J.D.C.; project administration, J.D.C.; funding acquisition, J.D.C. All authors have read and agreed to the published version of the manuscript.

## Availability of data and materials

Not applicable

## References

1. Bansal, D.P., et al., *ARDS following inhalation of hydrochloric acid.* J Assoc Physicians India, 2011. **59**: p. 115-7.
2. Boyce, S.H. and K.A. Simpson, *Hydrochloric acid inhalation: who needs admission?* J Accid Emerg Med, 1996. **13**(6): p. 422-4.
3. Heidari, H., et al., *Respiratory effects of occupational exposure to low concentration of hydrochloric acid among exposed workers: a case study in steel industry.* Med Gas Res, 2019. **9**(4): p. 208-212.
4. Roslan, N.L., K. Urgena, and M.A. Wahab, *The Toxic Fumes: A Case Report of an Accidental Inhalation of HCL 33%.* Asia Pacific Journal of Medical Toxicology, 2023. **12**(1): p. 33-37.
5. Chen, Y., et al., *Exposure to occupational risk factors is associated with the severity and progression of chronic obstructive pulmonary disease.* Medicine, 2023. **102**(6).

6. *Occupational exposures to mists and vapours from strong inorganic acids and other industrial chemicals. Working Group views and expert opinions, Lyon, 15-22 October 1991.* IARC Monogr Eval Carcinog Risks Hum, 1992. **54**: p. 1-310.
7. Cohen, M.L., et al., *A fibroblast-dependent TGFβ1/sFRP2 noncanonical Wnt signaling axis underlies epithelial metaplasia in idiopathic pulmonary fibrosis.* bioRxiv, 2023.
8. Basoalto, R., et al., *Acute lung injury secondary to hydrochloric acid instillation induces small airway hyperresponsiveness.* Am J Transl Res, 2021. **13**(11): p. 12734-12741.
9. Burleigh-Flayer, H., K.L. Wong, and Y. Alarie, *Evaluation of the pulmonary effects of HCl using CO2 challenges in guinea pigs.* Fundam Appl Toxicol, 1985. **5**(5): p. 978-85.
10. Marinova, M., et al., *Acute exposure of mice to hydrochloric acid leads to the development of chronic lung injury and pulmonary fibrosis.* Inhal Toxicol, 2019. **31**(4): p. 147-160.
11. Oddoy, A., et al., *[The effect of intermittent hydrogen chloride exposure on the lung function of the guinea pig].* Z Erkr Atmungsorgane, 1982. **158**(3): p. 285-90.
12. Westervelt, C.L., et al., *Effects of anti-inflammatory agents on hydrochloric acid-induced pulmonary injury.* J Invest Surg, 1996. **9**(4): p. 283-91.
13. Kamaruzaman, N.A., et al., *The Rabbit as a Model for Studying Lung Disease and Stem Cell Therapy.* BioMed Research International, 2013. **2013**: p. 691830.
14. Shampain, M.P., et al., *An animal model of late pulmonary responses to Alternaria challenge.* Am Rev Respir Dis, 1982. **126**(3): p. 493-8.
15. Kamaruzaman, N.A., et al., *The rabbit as a model for studying lung disease and stem cell therapy.* Biomed Res Int, 2013. **2013**: p. 691830.
16. Kobayashi, T., et al., *Lung lavage and surfactant replacement for hydrochloric acid aspiration in rabbits.* Acta Anaesthesiol Scand, 1990. **34**(3): p. 216-21.
17. Nishina, K., et al., *Intravenous Lidocaine Attenuates Acute Lung Injury Induced by Hydrochloric Acid Aspiration in Rabbits* Anesthesiology, 1998. **88**(5): p. 1300-1309.
18. Shellito, J. and S. Murphy, *The Effect of Experimental Acid Aspiration on Alveolar Macrophage Function in Rabbits.* American Review of Respiratory Disease, 1980. **122**(4): p. 551-560.
19. Phillips, J.E., *Inhaled efficacious dose translation from rodent to human: A retrospective analysis of clinical standards for respiratory diseases.* Pharmacol Ther, 2017. **178**: p. 141-147.
20. Henderson, Y.H., Howard W., *Noxious gases.* Reinhold Publishing Corporation, New York, 1943, 1943: p. 294.
21. Forbes, B., et al., *Challenges in inhaled product development and opportunities for open innovation.* Adv Drug Deliv Rev, 2011. **63**(1-2): p. 69-87.
22. Snipes, M.B., et al., *Retention patterns for inhaled particles in the lung: comparisons between laboratory animals and humans for chronic exposures.* Health Phys, 1989. **57 Suppl 1**: p. 69-77; discussion 77-8.

23. Ashcroft, T., J.M. Simpson, and V. Timbrell, *Simple method of estimating severity of pulmonary fibrosis on a numerical scale.* J Clin Pathol, 1988. **41**(4): p. 467-70.
24. Matute-Bello, G., et al., *An official American Thoracic Society workshop report: features and measurements of experimental acute lung injury in animals.* Am J Respir Cell Mol Biol, 2011. **44**(5): p. 725-38.
25. Wilson, M.S. and T.A. Wynn, *Pulmonary fibrosis: pathogenesis, etiology and regulation.* Mucosal Immunol, 2009. **2**(2): p. 103-21.
26. Todd, N.W., I.G. Luzina, and S.P. Atamas, *Molecular and cellular mechanisms of pulmonary fibrosis.* Fibrogenesis Tissue Repair, 2012. **5**(1): p. 11.
27. B, B.M., et al., *Animal models of fibrotic lung disease.* Am J Respir Cell Mol Biol, 2013. **49**(2): p. 167-79.
28. Liu, P., I.N. Zelko, and J. Yu, *A comparative study of bronchopulmonary slowly adapting receptors between rabbits and rats.* Physiol Rep, 2022. **10**(6): p. e15069.
29. Esteves, P.J., et al., *The wide utility of rabbits as models of human diseases.* Exp Mol Med, 2018. **50**(5): p. 1-10.
30. Sadeghi, A., et al., *Pharmacokinetics of intravitreal macromolecules: Scaling between rats and rabbits.* European Journal of Pharmaceutical Sciences, 2021. **159**: p. 105720.
31. Churg, A. and J.L. Wright, *Bronchiolitis caused by occupational and ambient atmospheric particles.* Semin Respir Crit Care Med, 2003. **24**(5): p. 577-84.
32. Weber, J., H. Peng, and C. Rader, *From rabbit antibody repertoires to rabbit monoclonal antibodies.* Experimental & Molecular Medicine, 2017. **49**(3): p. e305-e305.
33. Dodd, D.C., et al., *Experimental acid-aspiration pneumonia in the rabbit. A pathologic and morphometric study.* Vet Pathol, 1976. **13**(6): p. 436-48.
34. Folkesson, H.G., et al., *Acid aspiration-induced lung injury in rabbits is mediated by interleukin-8-dependent mechanisms.* The Journal of Clinical Investigation, 1995. **96**(1): p. 107-116.
35. Meltzer, E.B. and P.W. Noble, *Idiopathic pulmonary fibrosis.* Orphanet J Rare Dis, 2008. **3**: p. 8.
36. Solopov, P., et al., *Dietary Phytoestrogens Ameliorate Hydrochloric Acid-Induced Chronic Lung Injury and Pulmonary Fibrosis in Mice.* Nutrients, 2021. **13**(10).
37. Solopov, P., et al., *Development of chronic lung injury and pulmonary fibrosis in mice following acute exposure to nitrogen mustard.* Inhalation Toxicology, 2020. **32**(4): p. 141-154.
38. Herberts, M.B., et al., *Idiopathic pulmonary fibrosis in the United States: time to diagnosis and treatment.* BMC Pulmonary Medicine, 2023. **23**(1): p. 281.
39. Snyder, L.D., et al., *Time to diagnosis of idiopathic pulmonary fibrosis in the IPF-PRO Registry.* BMJ Open Respir Res, 2020. **7**(1).
40. Kopitar, Z., et al., *Species differences in metabolism and excretion of bromhexine in mice, rats, rabbits, dogs and man.* European Journal of Pharmacology, 1973. **21**(1): p. 6-10.

41. Buechler, M.B., W. Fu, and S.J. Turley, *Fibroblast-macrophage reciprocal interactions in health, fibrosis, and cancer.* Immunity, 2021. **54**(5): p. 903-915.
42. Nouno, T., et al., *Elevation of pulmonary CD163+ and CD204+ macrophages is associated with the clinical course of idiopathic pulmonary fibrosis patients.* Journal of Thoracic Disease, 2019. **11**(9): p. 4005-4017.
43. Chauvin, P., et al., *Soluble CD163 is produced by monocyte-derived and alveolar macrophages, and is not associated with the severity of idiopathic pulmonary fibrosis.* Innate Immun, 2022. **28**(3-4): p. 138-151.
44. Bibaki, E., et al., *CD163 positive alveolar macrophages from bronchoalveolar lavage in idiopathic pulmonary fibrosis and lung cancer.* European Respiratory Journal, 2019. **54**(suppl 63): p. PA1306.
45. Beamer, C.A. and A. Holian, *Scavenger receptor class A type I/II (CD204) null mice fail to develop fibrosis following silica exposure.* Am J Physiol Lung Cell Mol Physiol, 2005. **289**(2): p. L186-95.
46. Sontake, V., et al., *Hsp90 regulation of fibroblast activation in pulmonary fibrosis.* JCI Insight, 2017. **2**(4): p. e91454.
47. Colunga Biancatelli, R.M.L., et al., *The Heat Shock Protein 90 Inhibitor, AT13387, Protects the Alveolo-Capillary Barrier and Prevents HCl-Induced Chronic Lung Injury and Pulmonary Fibrosis.* Cells, 2022. **11**(6).
48. Colunga Biancatelli, R.M.L., et al. *The HSP90 Inhibitor, AUY-922, Protects and Repairs Human Lung Microvascular Endothelial Cells from Hydrochloric Acid-Induced Endothelial Barrier Dysfunction*. Cells, 2021. **10**, 1489 DOI: 10.3390/cells10061489.
49. Plantier, L., et al., *Physiology of the lung in idiopathic pulmonary fibrosis.* European Respiratory Review, 2018. **27**(147): p. 170062.
50. Solopov, P.A., et al., *Optimizing antidotal treatment with the oral HSP90 inhibitor TAS-116 against hydrochloric acid-induced pulmonary fibrosis in mice.* Frontiers in Pharmacology, 2022. **13**.
51. Han, M.K., et al., *Sex differences in physiological progression of idiopathic pulmonary fibrosis.* European Respiratory Journal, 2008. **31**(6): p. 1183.
52. Ozaki, M., et al., *Sexual Dimorphism in Interstitial Lung Disease.* Biomedicines, 2022. **10**(12).
53. Sesé, L., et al., *Gender Differences in Idiopathic Pulmonary Fibrosis: Are Men and Women Equal?* Front Med (Lausanne), 2021. **8**: p. 713698.
54. Solopov, P., et al., *Sex-Related Differences in Murine Models of Chemically Induced Pulmonary Fibrosis.* Int J Mol Sci, 2021. **22**(11).

## Figures

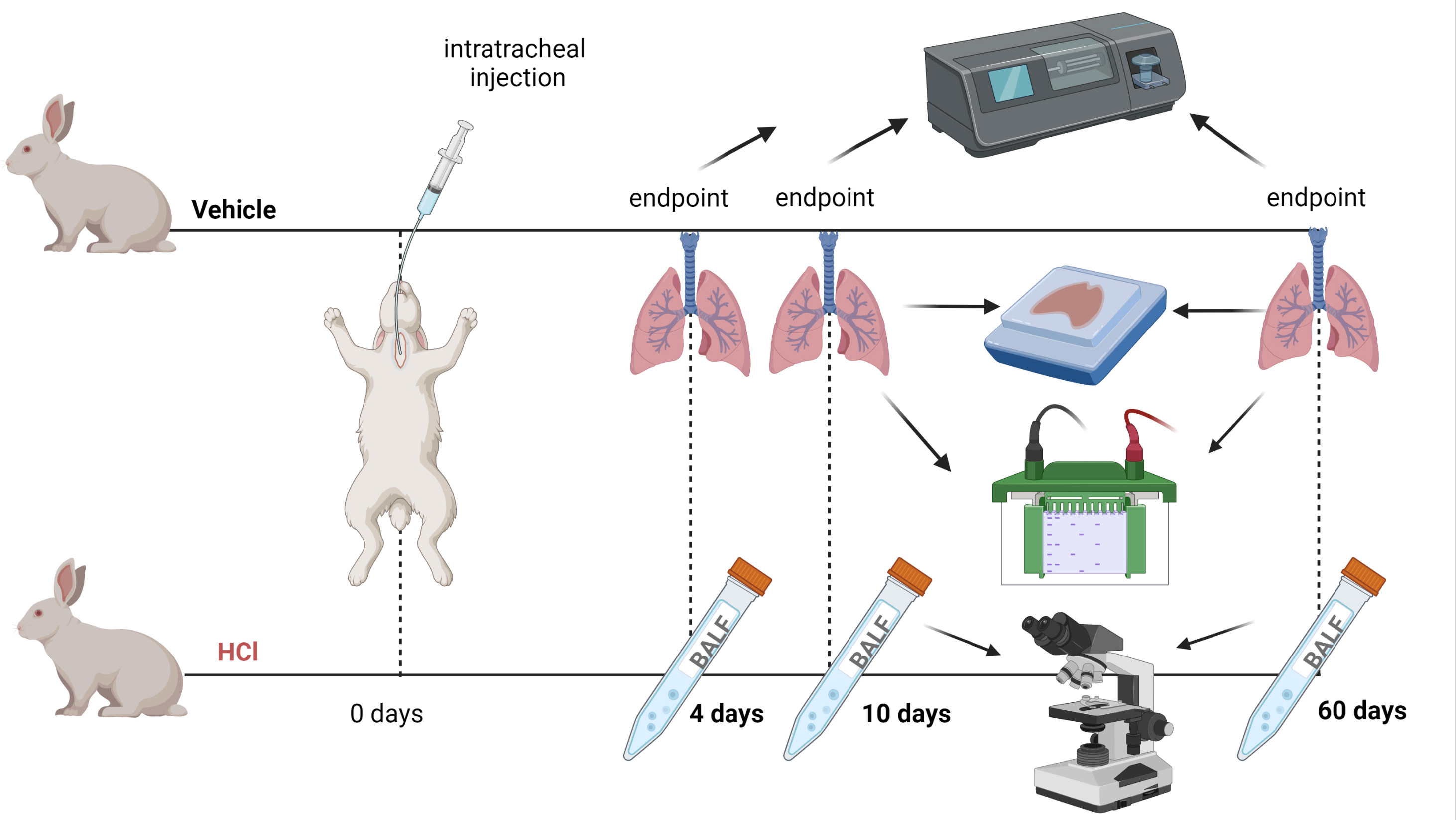

### Figure 1

**Experimental design**. *Rabbits were injected with HCl or saline, intratracheally on day 0. Bronchoalveolar Lavage Fluid (BALF) was collected and analyzed in one set of animals (n=7/treatment group/time point). Lung function (Flexi-Vent, n=4-6/treatment group/time point), and histology (n=7/treatment group/time point) studies were performed using separate sets of animals.*

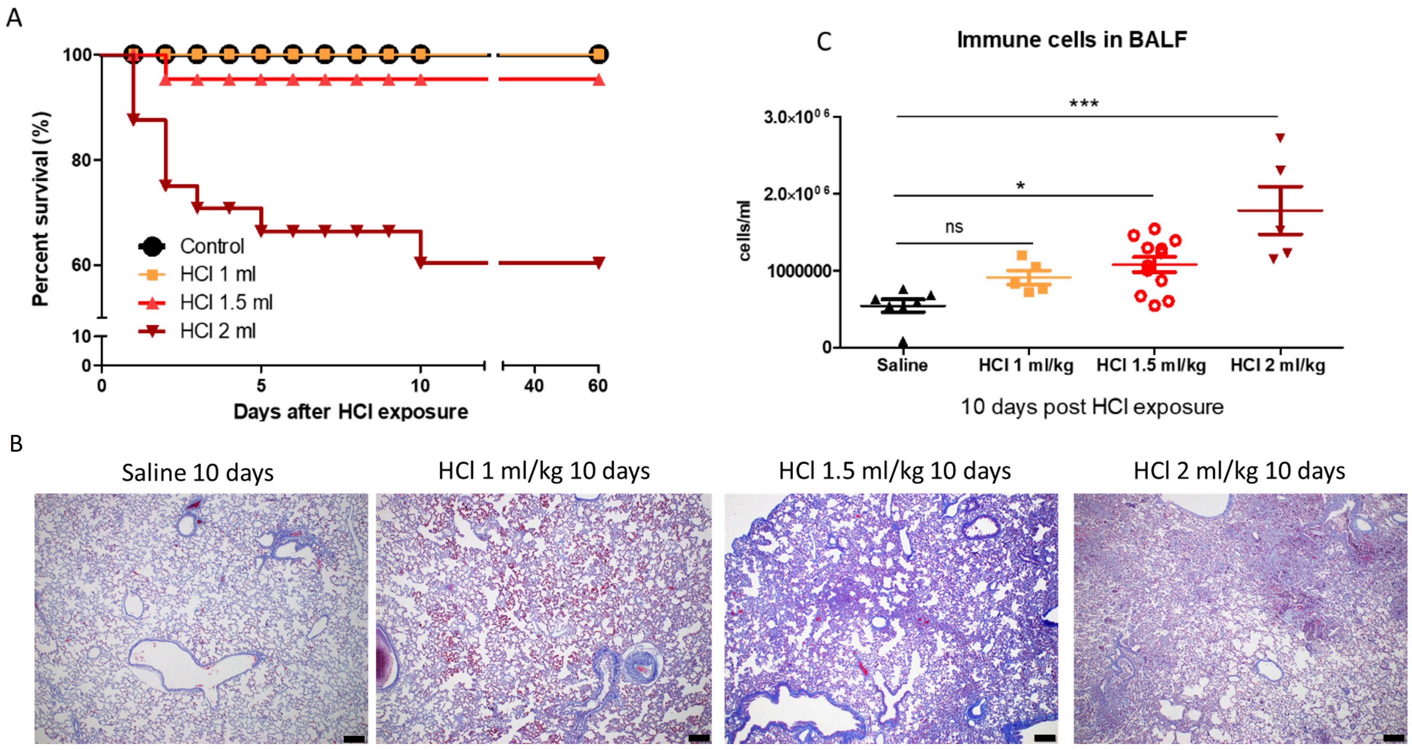

### Figure 2

***Survival, Alveolar Inflammation, and Lung Histological Changes in Response to Different Doses of HCl.*** *Rabbits were injected intratracheally with 0.1N HCl at 1, 1.5 or 2 ml/kg.* ***A:*** *Rabbits injected with 1 or 1.5 ml/kg demonstrated high survival rate, while 2 ml/kg dose provoked significant mortality; Kaplan-Meier survival graph, n=10-20/group.* ***B****: Masson's trichrome staining of lung sections 10 d after HCl or Saline. Rabbits, exposed to 1.5 and 2 ml/kg of 0.1N HCl show strong alveolar inflammation and change of parenchymal lung architecture, while rabbits injected with 1 ml/kg of HCl or Saline have no significant changes.* ***C:*** *Rabbits, injected with 1.5 and 2 mg/kg demonstrated significant increase in bronchoalveolar lavage fluid immune cells compared to controls; n=7-10/group, ***p<0.001; *P<0.05; ns – not significant, with ANOVA and Tukey's post-hoc test.*

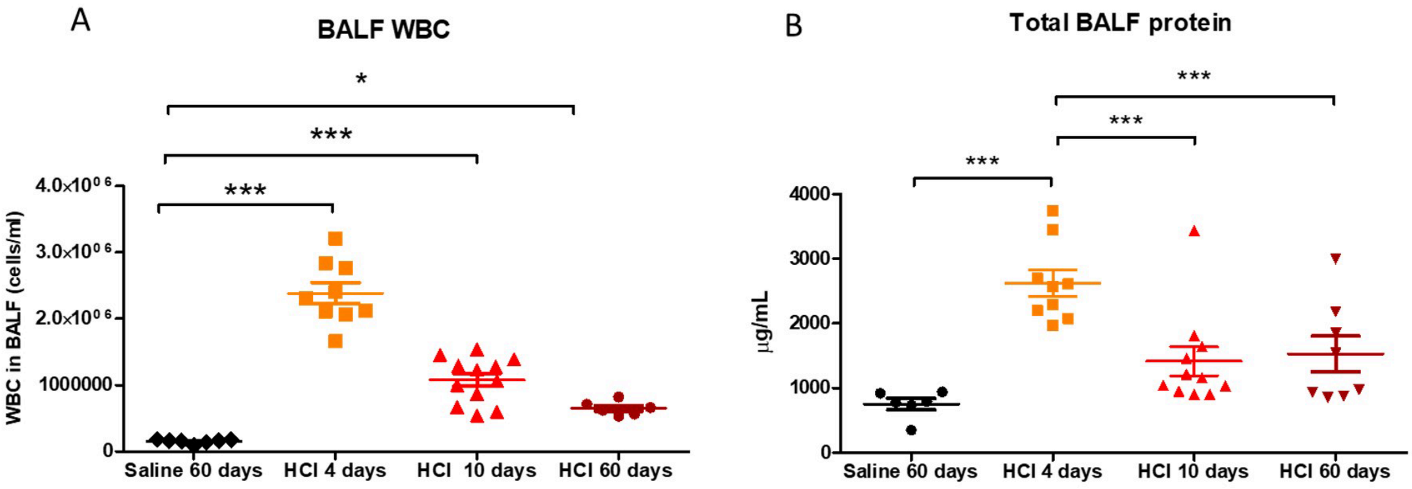

### Figure 3

***Elevations in immune cell number (A) and total protein content (B) in bronchoalveolar lavage fluid of rabbits 4, 10 or 60 days after 1.5 ml/kg 0.1N HCl or Saline intratracheal instillation.*** ****p<0.001; *P<0.05 with ANOVA and Tukey's post-test; n=7-12/group.*

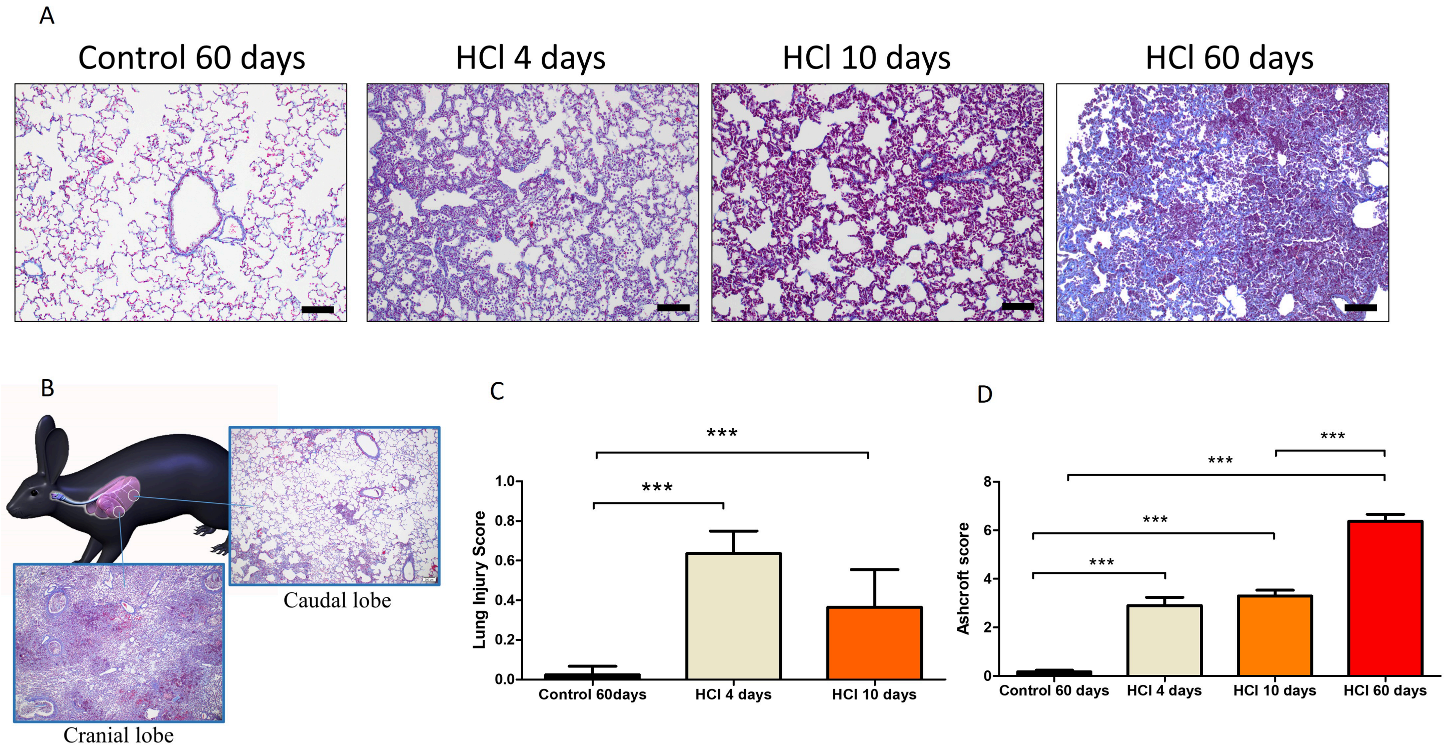

### Figure 4

***Masson's trichrome staining of rabbit lung sections at 4, 10, and 60 days after HCl or 60 days post Saline intratracheal instillation****. A: time dependent pathohistological changes; B: differences between HCl-induced chronic injuries in cranial and caudal lobes 60 days post HCl; C: Lung Injury Scoring of histological slides collected from rabbits 4- and 10-days post HCl or Saline 60 days; D: Ashcroft score of lung fibrosis 4-, 10- and 60-days after HCl instillation. Data are means ± SEM, ***: p < 0.001 compared to saline-control rabbits with ANOVA and Tukey's post-test. Original magnification: 20×, scale bar: 100μm.*

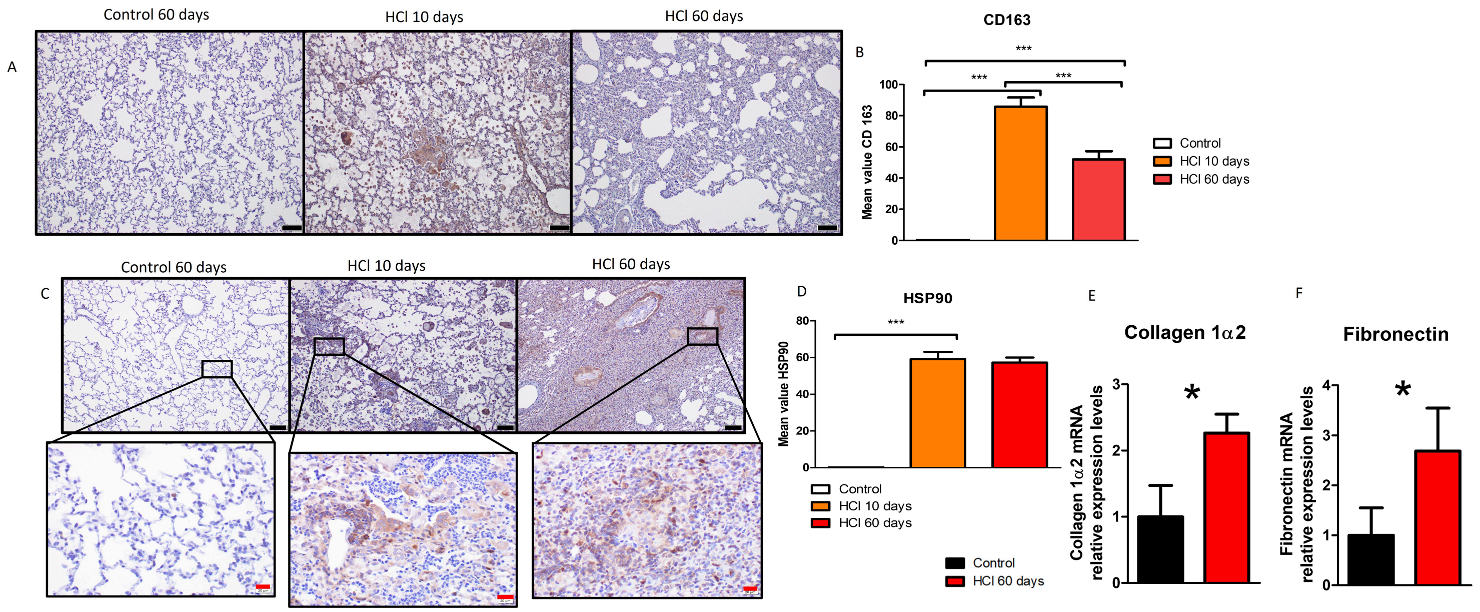

### Figure 5

***Immunostaining and quantification analysis of rabbit lung tissue.*** *Expression of CD163 in lung tissue after saline or HCl instillation (A) and semi-quantitative determination of CD163 expression (B). Expression of HSP90β in lung tissue after saline or HCl instillation (C) and semi-quantitative determination of HSP90 (D). Relative expression levels of (E) Collagen 1α2 and Fibronectin (F) 60 days after either saline or HCl 0.1N 1.5 ml/Kg exposure. Data are means ± SEM, n=3-5; ***: $p < 0.001$ compared to saline-control rabbits with ANOVA and Tukey's post-hoc test. Original magnification: 20×, scale bar: 100μm.*

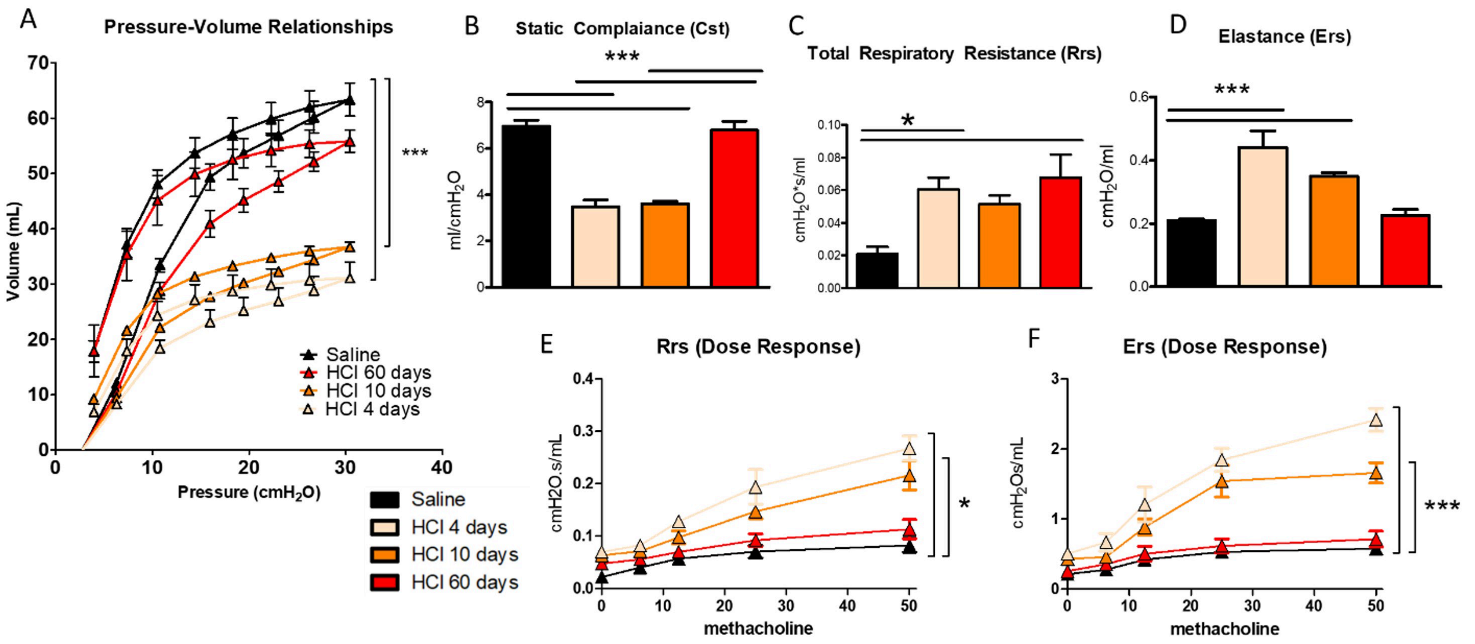

### Figure 6

***Changes in respiratory mechanics at 4, 10 and 30 days after instillation of 1.5ml/kg 0.1N HCl.*** *Pressure-volume loops (A), Static Compliance (Cst) (B), total respiratory system resistance (Rrs) (C) and total elastance (Ers) (D) were evaluated in response to increasing concentrations of aerosolized methacholine in vehicle- and HCl-treated rabbits. Mean ±SEM (n=4-6) ***p<0.001, *p<0.05 compared to Saline curve. with ANOVA and Tukey's post-hoc test.*

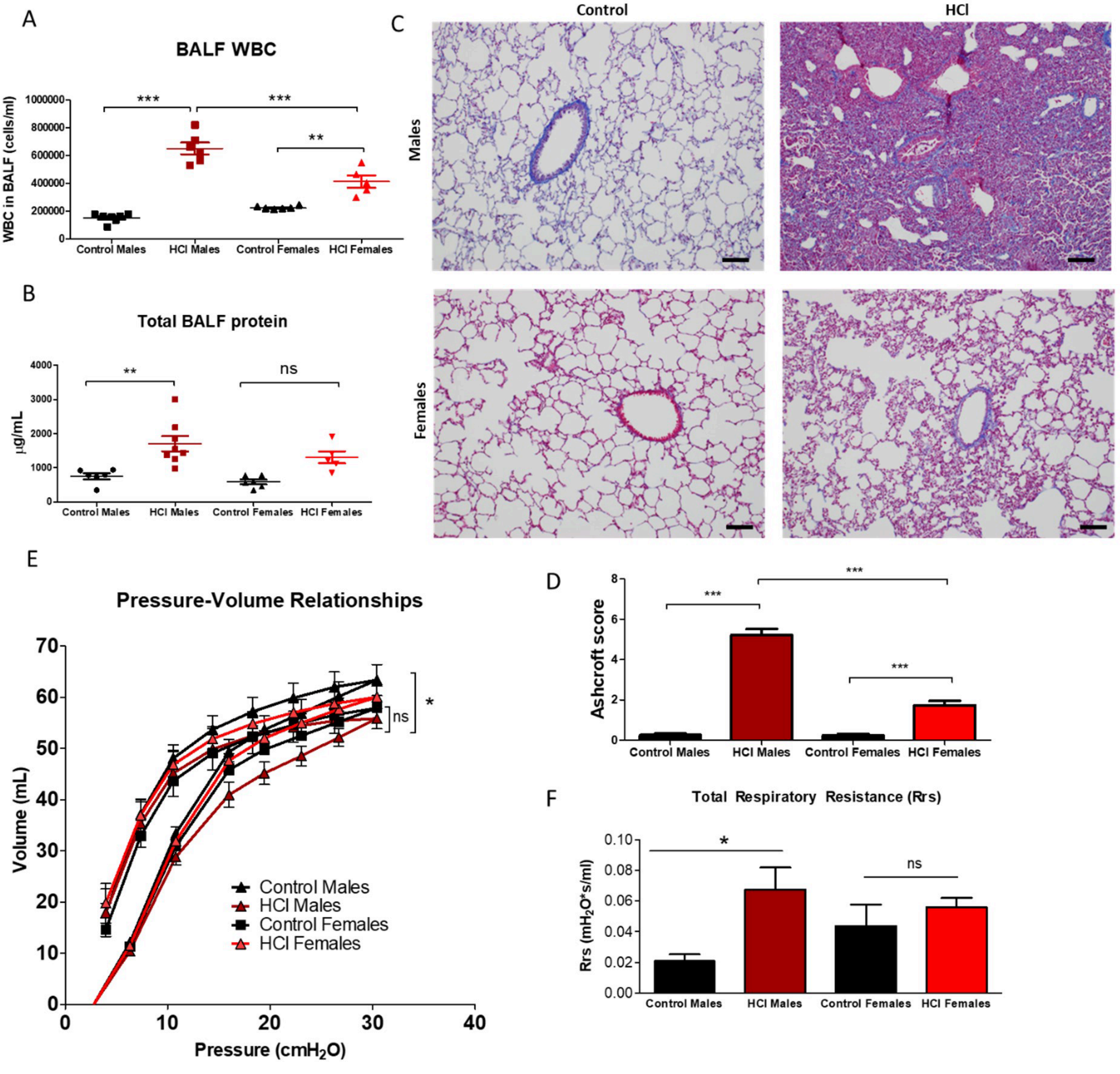

Figure 7

***Sex differences in rabbits exposed to 1.5 ml/kg 0.1N HCl.*** *A: Immune cells content in BALF; B: Total protein concentration in BALF; C: Masson's trichrome staining of lung sections and Ashcroft scoring (D);*

*E: Pressure-volume loops; F: Total respiratory resistance (Rrs). *p<0.05, **p<0.01, ***p<0.001, ns – not significant, compared to Saline-injected rabbits. with ANOVA and Tukey's post-hoc test.*
